# Clinician approaches to communicating a dementia diagnosis: An interview study

**DOI:** 10.1371/journal.pone.0267161

**Published:** 2022-04-14

**Authors:** Easton N. Wollney, Carma L. Bylund, Noheli Bedenfield, Monica Rosselli, Rosie E. Curiel-Cid, Marcela Kitaigorodsky, Ximena Levy, Melissa J. Armstrong

**Affiliations:** 1 Department of Health Outcomes and Biomedical Informatics, University of Florida College of Medicine, Gainesville, FL, United States of America; 2 Department of Medicine, University of Florida College of Medicine, Gainesville, FL, United States of America; 3 Department of Neurology, University of Florida College of Medicine, Gainesville, FL, United States of America; 4 Department of Psychology, Florida Atlantic University, Davie, FL, United States of America; 5 Department of Psychiatry & Behavioral Sciences, University of Miami Miller School of Medicine, Miami, FL, United States of America; 6 Clinical Research Unit, Division of Research, Florida Atlantic University, Boca Raton, FL, United States of America; Nathan S Kline Institute, UNITED STATES

## Abstract

**Background:**

Individuals with cognitive impairment and their families place a high value on receiving a dementia diagnosis, but clinician approaches vary. There is a need for research investigating experiences of giving and receiving dementia diagnoses. The current study aimed to investigate clinician approaches to giving dementia diagnoses as part of a larger study investigating patient, caregiver, and clinician experiences during the diagnosis encounter.

**Method:**

Investigators conducted telephone interviews with Florida-based clinicians who give dementia diagnoses either rarely or commonly. Interviews employed a semi-structured interview guide querying communication practices used by clinicians when giving dementia diagnoses and how clinicians learned to give dementia diagnoses. Investigators used a descriptive qualitative design to conduct a thematic analysis of data.

**Results:**

Fifteen Florida-based clinicians participated, representing diverse backgrounds related to gender, race/ethnicity, specialty, and practice setting. Participants reported using patient- and family-centered communication practices including checking patient understanding, communicating empathically, and involving family members. Some clinicians explicitly asked patients and/or family members about their preferences regarding diagnosis disclosure; many clinicians tailored their disclosure based on patient and family characteristics or reactions. Some clinicians reported using specific diagnoses, while others used general terms such as “memory disorder.” Clinicians reported positively framing information, including instilling hope, focusing on healthy behaviors, and discussing symptom management. Finally, clinicians provided patient/family education and arranged follow up. Clinicians reported learning approaches to dementia diagnosis disclosure through formal training and self-education.

**Conclusions:**

Diverse Florida-based clinicians described dementia disclosure practices largely consistent with published guidance, but clinicians varied on approaches relating to soliciting patient disclosure preferences and terminology used. Clinicians caring for diverse populations described that cultural background affects the disclosure process, but more research is needed regarding this finding and best practices for individuals from different backgrounds.

## Introduction

The proportion of individuals who receive a diagnosis of dementia has increased worldwide [1] due in part to greater awareness and acceptance of dementia. Individuals with cognitive impairment and their families place a high value on understanding a dementia diagnosis and knowing the specific etiology (e.g., Alzheimer disease [AD]) [2]. They describe a “right to know” their diagnosis [3, 4] and express benefits of receiving a formal (i.e., specific) diagnosis including understanding what is happening [3], validation that something is wrong [5, 6], improved family patience with the person with dementia [5], obtaining appropriate medical management and connecting the individual and their family to resources [3, 6], and options to participate in clinical trials [7]. Based on some of these benefits, dementia diagnosis disclosure can result in improved quality of life (QoL), decision-making and future-planning [3–5, 8], as well as reduce anxiety for some patients and care partners [9].

Though individuals with dementia or cognitive impairment have expressed a preference to know their diagnosis and many benefits are identified, research suggests that clinicians often do not disclose diagnoses of AD or AD-related dementias (ADRDs). The Alzheimer’s Association found that only 45% of individuals living in the U.S. with symptomatic AD and 27% of individuals with other dementia-related illnesses received a diagnosis [10]. A systematic review of research regarding practices for communicating a diagnosis of dementia identified that only 34% of primary care physicians and 48% of specialists routinely tell the person with dementia their diagnosis [11]. Revealing the diagnosis to care partners/caregivers is more common– 89% of primary care physicians and 97% of specialists give a diagnosis to care partners/caregivers [11]. When this topic is discussed, some clinicians use euphemistic terms such as “memory problems” rather than formal diagnoses [11–13].

Reasons for non-disclosure are complex and likely relate to patient-, caregiver-, and clinician-level factors. Interactions between clinicians, patients, and family members are made more challenging due to the presence of cognitive impairment [14–16] and the involvement of a third person in the interaction such as a family caregiver (i.e., the dementia triad) [14, 15, 17, 18]. This can be both helpful and unhelpful for clinicians [18, 19]. For example, it may be helpful when the third person can help to facilitate patient understanding or provide support for the patient [19] but may be unhelpful when the third person engages in frequent interruptions [19, 20] or has preferences that conflict with the patient’s preferences [18, 19]. Other ecological issues also create barriers to receiving or giving a diagnosis, such as limited access to specialist or support services [16]. In the U.S., clinicians have reported a lack of local dementia specialists to meet demands, which means primary care physicians (PCPs) are often responsible for diagnosing and treating dementia [16]. Yet PCPs may be insufficiently trained to properly make and communicate the clinical diagnosis to individuals with cognitive impairment and their families [7, 21]. Only about 60% of PCPs in one survey reported feeling fully comfortable diagnosing dementia [16]. Clinicians’ low confidence in their ability to diagnose dementia has also been found in other studies [12, 21, 22]. This may be associated with limited training in this area, as about 65% of primary care physicians reported “very little” and 25% reported having no residency training in dementia diagnosis and care [16].

Additional reasons for clinician reluctance to make or disclose dementia diagnoses have been identified and may include clinicians not wanting to give a diagnosis that isn’t definite [12], perceived lack of benefit of an early diagnosis [21, 23], concerns regarding emotional distress associated with the diagnosis [23, 24], the perception that the patient does not want to receive a diagnosis [12, 23] or will be unable to understand the diagnosis [23, 24], therapeutic nihilism [21–24], and concerns about dementia stigma [12, 21–24]. Clinicians may also believe that costs of delivering a diagnosis outweigh benefits [23, 24], there is insufficient remuneration for provision of dementia care [22], or that diagnosis will strain medical systems [21, 23].

Challenges in giving a dementia diagnosis may be further exacerbated by cultural or language barriers, although triadic barriers seem to exist across cultures. In a 2018 study using interviews to assess the process of diagnosing dementia in immigrant groups, clinicians described challenges in assessing and diagnosing dementia due to limited experience, language barriers, lack of continuity, and cross-cultural differences [25]. Primary care physicians in Japan described choices in dementia diagnosis disclosure including who they target (patient and/or care partner), clarity of diagnosis and prognosis (disclose clearly, use euphemistic terms, versus no disclosure), and case-specific disclosure adjustments [26]. Primary care physicians who were highly comfortable with the disclosure process were more likely to discuss the diagnosis with both patients and families, whereas physicians who were uncomfortable tended to refrain from giving dementia diagnoses and emphasized negative aspects of dementia [26].

Although barriers to disclosing a dementia diagnosis have been identified, several gaps in the literature still exist. First, studies have often described barriers to dementia disclosure, but less is known about other aspects of the disclosure interaction (e.g., clinician recommendations or current strategies used) [27]. Second, studies that have examined the disclosure interaction more broadly (i.e., not just barriers to communicating a diagnosis) have often focused on clinical observations [18, 20]. While this is helpful in describing aspects of the interaction, clinicians’ perspectives may be missing. For example, a 2012 study analyzed real-time encounters involving six clinicians and found that diagnosis disclosure involved a series of alternating dyadic exchanges (physician-patient, physician-caregiver), but did not query clinicians regarding approaches or experiences [20]. Similar research assessed patient and companion experiences of diagnosis disclosure through post-encounter interviews but did not interview the participating clinicians [6]. A 2013 systematic review found that most studies assessing clinicians’ perspectives on disclosing a diagnosis were quantitative (e.g., mail surveys) [27]. This review, as well as a more recent review [2], concluded that more research was needed regarding dementia diagnosis disclosure to better understand what health professionals should bring to the diagnosis revelation process.

Given limited recent research describing clinicians’ experiences disclosing a dementia diagnosis to individuals and family members, this study sought to better understand the diagnosis disclosure process from clinicians’ perspectives including individuals with diverse backgrounds. The study started with research questions (RQs) regarding clinician experiences in (1) giving a dementia diagnosis and (2) learning how to give a dementia diagnosis (to better understand how clinicians developed their communication practices):

RQ1: What communication practices do clinicians report using when giving a dementia diagnosis to individuals with dementia and their families?
RQ2: How did clinicians learn to give a dementia diagnosis?

## Methods

This study is the first step in a larger, ongoing, multi-stage study to investigate the experiences of giving and receiving dementia diagnoses among three groups: clinicians, individuals with AD or an ADRD, and caregivers. Methods are reported using the Consolidated criteria for Reporting Qualitative Research (COREQ) Checklist [28] (S2 File).

### Approach

We used a descriptive qualitative design [29, 30] through telephone interviews to investigate clinicians’ self-reported experiences when communicating a diagnosis of dementia to individuals with AD/ADRD and their families. Qualitative methodologies allow for a deeper understanding of the complex communication dynamics between health professionals, individuals, and their families. Furthermore, qualitative methodologies enable a deeper and stronger understanding of clinical-social problems [31, 32]. Semi-structured interviews were chosen for the current study to allow clinicians to reflect on their overall experiences across patients, optimal approaches to communication, and methods by which they developed their communication strategies; information that is not obtainable through other study designs (e.g., recording of actual clinical encounters).

### Population and recruitment

Participants were Florida-based clinicians who self-reported giving diagnoses of dementia (rarely or frequently). Recruitment was limited to clinicians in Florida given that the study was funded by the Florida Department of Health Ed & Ethel Moore Alzheimer’s Disease Research Program. In the U.S., Florida is second only to California for the number of residents 65 years and older diagnosed with AD (580,000). The number of individuals living with AD in Florida is expected to increase by 24% between years 2020–2025 [16].

We used stratified sampling [33] to recruit clinicians based on specialty/sub-specialty, location within Florida, and years in practice. We recruited participants through the professional networks of investigators at the three study sites (University of Florida, University of Miami, Florida Atlantic University), as well as the state of Florida memory disorders clinics, advertising through Florida Medical Association and the Florida Dementia Care and Cure Initiative task force mailings, and Facebook advertising designed by the University of Florida Clinical and Translational Science Institute. Potential participants were approached via email, in person, or online. Those who were potentially interested in participating contacted the study coordinator by email or phone to learn more about the study and schedule a one-time telephone interview. All procedures were approved by the University of Florida Institutional Review Board (IRB202000212). We used a waiver of documentation of informed consent, which was shared with participants in advance. At the beginning of the telephone interview, participants had the opportunity to ask questions about the information in the waiver. Participants were eligible to receive a $25 gift card after completing the interview.

### Data collection

Three authors (ENW, CLB, MJA) developed the semi-structured interview guide (S1 File) based on the healthcare communication literature. Semi-structured interview guides allow for greater flexibility for both the interviewer and participant than structured interviewing but provide greater direction than unstructured interviews [33]. The lead investigators represented expertise in both healthcare communication and dementia. The interview guide had 10 questions about communicating a diagnosis, with possible probes for appropriate questions (e.g., “To whom do you generally give the diagnosis to (patient/caregiver)? If it varies, how do you make that decision?”). Additionally, clinicians were asked about their clinical background (e.g., training, specialty, and years in practice) and other demographic information such as age, gender, and race/ethnicity. The first author (ENW), a PhD student in healthcare communication at the time of the interviews with training in qualitative methods, conducted all interviews by phone. She had no prior relationship with any of the participants. Interviews were conducted in English and occurred between 6/4/20 and 2/22/21. All telephone interviews were audio recorded and subsequently professionally transcribed and de-identified. Interview notes were taken by the first author (ENW) during interviews and securely stored. Participant checking was not performed.

### Data analysis

We used a thematic analysis method to analyze and present data through an inductive process in which patterns, or themes, emerged from the data, with no existing framework or theoretical constructs used to classify data [29, 34]. We used Braun & Clarkes’ [29] criteria for thematic analysis, which includes an iterative process such as starting with broad codes, collapsing codes, and continuous refinement until themes are finalized and contain rich descriptions [35]. This process contains several overlapping features of the constant comparative method, which is commonly used in grounded theory [29, 36–38]. However, thematic analysis does not require theory development [29]. To ensure rigor, we used an iterative process between design and analysis [39]. We considered themes to have a strong degree of saturation when no new codes emerged from the data, utilizing an inductive thematic approach to saturation [40].

To begin, two coders (ENW, CLB) familiarized themselves with the data and open-coded transcripts for the two research questions to label and develop an initial coding scheme. A codebook was then created and subsequently refined several times throughout this process. Codes were then collapsed into broader categories representing related concepts and overarching themes [29]. After overarching themes were developed, the primary coder (ENW) closed-coded all 15 transcripts to further establish thematic descriptions and identify thematic exemplars in the codebook [29, 37]. After codes were collapsed and themes were formed, an additional coder (CLB) provided verification by close-coding 5 transcripts. The codebook was then shared with two additional authors (MJA and MR) as a further verification, including for cultural fairness. Memos were kept and securely stored throughout the coding process by the primary coder (ENW) to develop and refine thematic descriptions [39, 41]. Throughout data collection and analysis, the primary study team met weekly to discuss the data, including emerging thematic categorization and properties. We managed the qualitative data using ATLAS.ti software.

## Results

### Participant characteristics

Fifteen clinicians with different backgrounds participated in interviews (Table 1). Of the five individuals with a primary focus in family medicine/geriatrics, two trained solely in family medicine, one trained in internal medicine with a geriatrics fellowship, one trained in family medicine with additional training in geriatrics, and one trained as a geriatric nurse practitioner with additional certification in psychiatry. This was the only non-physician participant with specialties in family medicine/geriatrics or neurology. Of the neurologists, one had additional training in palliative care and two described a particular focus on behavioral neurology/memory disorders. The participating psychiatrist was trained in general and geriatric psychiatry. Eleven of the participants were associated with an academic medical center. Because of the range in specialties and training, some clinicians provided on-going care to patients after making a diagnosis, while others primarily engaged in a single consultation (e.g., to make and disclose the diagnosis), and some clinicians engaged in both types of interactions. Average interview duration was 25 minutes, 22 seconds (range 14 minutes, 13 seconds to 43 minutes, 24 seconds). Thirteen participants were native English speakers. The two Hispanic participants reported Spanish as their native language, but they were fluent Spanish/English bilinguals.

**Table 1:** Participant demographics.

Demographic Category	Clinician Demographics (n = 15)
Age (median, range) (years)	53 (34–82)
Gender (n, % male)	8 (53%)
Race (n, %)	
White non-Hispanic	11 (73%)
White Hispanic	2 (13%)
African American	1 (7%)
Asian	1 (7%)
Years in practice (median, range)	23 (2–50)
Primary specialty (n, %)	
Neurology (MD)	5 (33%)
Neuropsychology	3 (20%)
Family medicine/geriatrics	5 (33%)
Psychiatry	1 (7%)
Social work	1 (7%)

### Clinicians’ current practices

Five major themes emerged to answer the first research question regarding clinicians’ current practices used to communicate a dementia diagnosis: (1) using patient- and family-centered communication; (2) tailoring communication to the patient/family; (3) using deliberate choice in diagnostic terms; (4) positively framing information related to the diagnosis; and (5) providing patient and family education. Properties that comprise each theme are presented in italics. Quotes are referenced using the study participant ID.

#### Using patient- and family-centered communication

First, to facilitate patient and family centered communication, clinicians described using practices that reflected general recommendations for good interpersonal health communication across a variety of medical contexts. These practices included involving family members, checking patient and family understanding, and communicating empathically.

Clinicians discussed *involving family members* when delivering a dementia diagnosis to build rapport with the family. Clinicians may also engage family members to solicit information regarding the patient. While some clinicians discussed talking to patients and family members together, others reported arranging time alone with the family. For example, one clinician described conducting their initial consultation with patients and family members together before talking to each person separately, which helped better understand the individual needs of the patient and their family members, as needs are often different: “Because of [the initial consultation] and because of my one-on-one with the family during that first day, we create a plan with a family member on how to proceed in the case that the patient does have a dementia diagnosis” (05).

Clinicians reported *checking patient and family understanding* by asking if they understood the diagnosis and information being communicated, though not all clinicians used the same approaches (e.g., the teach-back method). For example, one clinician described asking the patient or family member to confirm that they understand the information being communicated: “Well, normally what I do is I asked them, ‘Do you understand what it is that I’m saying to you?’ I don’t ask them to repeat it back, but I ask them ‘Do you understand’” (03)? However, other clinicians asked patients to explicitly repeat back information: “l always ask them, ‘Tell me in your own words, what is your understanding of what I am saying’” (13)?

Furthermore, *communicating empathically* was described by clinicians as using sensitivity toward the challenges brought on by a dementia diagnosis and understanding the emotions that patients and family members may be experiencing upon receiving a diagnosis. This includes feeling overwhelmed following a diagnosis or preparing to make difficult decisions for the future. For instance, one clinician described their outlook as such:

I try to be as empathic as possible. There’s always the awareness among everybody in the room that if we’re making a dementia diagnosis, or making a diagnosis of a fundamentally untreatable disease, it’s going to make life for all involved harder and harder, [and] ultimately involves really miserable experiences, like the transfer to institutional care, and so on. (14)

Some clinicians described how their experiences delivering bad news to patients in other contexts (e.g., palliative care or social work) helped them to develop skills related to empathic communication. Clinicians were then able to transfer and use those skills when delivering a dementia diagnosis.

#### Tailoring communication to the patient/family

Second, clinicians discussed tailoring their communication when delivering a dementia diagnosis, though different factors influenced how and when clinicians decided to tailor information. Clinicians described tailoring after explicitly asking patients or family members for their information preferences prior to giving the diagnosis, including the type or amount of information they would like to know. Furthermore, clinicians described tailoring prior to or during the interaction based on patient and family characteristics. Finally, clinicians described tailoring in the moment based on the patient and family member’s reaction.

Clinicians sometimes described *asking patients and/or family members about information preferences prior to giving the diagnosis*. While some clinicians described only asking the patient or their family members for information preferences before giving the diagnosis, others described purposefully asking both individuals for information preferences. For example, this clinician described asking both for preferences while prioritizing the patient’s preferences: “I ask the patient and I ask the family member. But I first ask the patient, ‘if you would happen to have a diagnosis of dementia, how do you want me to disclose that information to you, with you, with the family’” (05)?

When *tailoring based on patient and family characteristics*, clinicians take into consideration several socio-demographic and disease-related factors before or during the interaction, including the patient’s level of cognition and their ability to process the information; the type of family support the patient has; and the patient’s educational background, culture, and prior knowledge about dementia. For example, clinicians who care for Hispanic/Latino patients discussed possible language barriers, as well as observed stigmas about having dementia that are present among the Hispanic/Latino communities. A Spanish-speaking neuropsychologist who specializes in working with Hispanic/Latino populations described the cultural differences she has observed in stigma regarding AD, which she perceived as being more greatly stigmatized among Hispanic/Latino communities:

In my experience, when you see Hispanics, I think there’s a lot of stigma associated to a diagnosis of dementia, especially with Alzheimer’s disease. Also, there’s a lot of normalization of the symptoms/cognitive problems in this population. So, I have a lot of Hispanics that [say], ‘Oh, you know, that’s normal aging’‘… It’s expected and normalized, and not seen as a disease process. In those cases, I feel that they need more education in terms of telling them this is not normal, that this is not the way it’s supposed to be. There’s a disease process in the brain that it’s causing this. And I think that I do see the difference in culture regarding that. (13)

Another clinician who sees many Hispanic/Latino patients described accounting for a range of experiences when communicating a dementia diagnosis: “Culture, meaning not only the ethnic and racial, but what does the patient understands that the diagnosis means in terms of their background or experience. That also needs to be taken into consideration” (05).

Clinicians who used *tailoring information in the moment based on patient and family member reactions* described reacting to cues and feedback from patients and/or their family during the interaction. One way in which clinicians did this was by trying to gauge how much time to give patients after delivering a diagnosis before delivering any further information. This was a practice described as allowing the patient to have the time and opportunity needed to process the diagnosis, because, as one clinician said, “one size does not fit all … people react to it in very different ways, and what you have to do is give people space” (02). Others described using emotional cues during the interaction as indicators of how to tailor the type or amount of information:

I’ll just say, “I’m most worried that you have Alzheimer’s disease.” And I’ll usually just let that hang for a second … and look for emotion in the patient or the family and try to respond to any emotional cues, and then step-by-step provide any additional information. (12)

#### Using deliberate choice in diagnostic terms

Third, clinicians described differences in language used when communicating a diagnosis. As several clinicians pointed out, there is often confusion among patients and family members regarding differences between AD and other types of dementia. Furthermore, various amounts and types of uncertainty exist regarding the diagnosis. Therefore, using deliberate choice in diagnostic terms included both what language clinicians use (general or specific language), as well as why clinicians prefer to use certain terms when giving a diagnosis.

*General language* included vague terms such as “dementia” or “memory disorder”. Reasons for not wanting to use specific language differed, such as the clinician not feeling they have seen the patient long enough to give a more specific diagnosis, not specializing in dementia (e.g., physicians in family medicine), and not wanting to further distress or confuse the patient. For example, one clinician said, “If I feel like they’re really in complete denial, I may just mention that they have a memory disorder” (10). Other clinicians were concerned about additional stress associated with certain terms: “I will typically use the word dementia first, to say you have dementia rather than using the Alzheimer’s word because that seems, in my experience, to carry less baggage with it” (03).

In contrast, other clinicians discussed why it is important to incorporate *specific language* when communicating a diagnosis. One of the reasons given for using specific language was to reduce confusion among patients about their diagnosis, such as the differences between AD and other types of dementias: “When you think of dementia, people automatically think Alzheimer’s disease… . people confuse dementia for the underlying cause of the dementia" (02). Similarly, some clinicians pointed out that specific language can be used to communicate about clinical uncertainty regarding the etiology: “It’s impossible based on the evidence to really say 100% that a patient has Alzheimer’s disease dementia. However, we can definitely say that the clinical presentation is one that looks like a dementia of Alzheimer’s type” (05). Therefore, clinicians discussed the need to make this distinction clear to patients and family members. This includes communicating clearly about how the clinician arrived at the diagnosis:

Most of them have been told that, “This looks like dementia,” but they’re concerned because they haven’t been told what specific type it is, and since they don’t know what type, they’re [left] wondering…. But when we can be specific about the diagnosis and make it transparent to why we’ve arrived at that diagnosis, even though these days, dementia is fundamentally untreatable…. they at least have some certainty. And that means a lot to people. (14)

#### Positively framing information related to the diagnosis

Fourth, clinicians acknowledged that dementia is a progressive disease with no cure. However, clinicians described positively framing information when delivering the diagnosis by *instilling hope*, *focusing on healthy behaviors*, and *managing symptoms* in order to improve or maintain patient quality of life.

Clinicians spent time *instilling hope* by discussing proactive steps patients can take to slow the progression or maintain quality of life as opposed to focusing on the negative aspects of the diagnosis. Clinicians explained that although there is no cure, instilling hope may motivate patients and families to take proactive steps to potentially decrease the burdens associated with ADRD. As one clinician said:

I think the most important thing is not to have the sort of nihilistic perspective that some people have, where you say, “Oh, you’ve got a degenerative condition and there’s nothing we can do about it, and there’s no cure” … My perspective has always been that you have to have people be hopeful, that you have to have people realize that there are things they can do that can also improve their cognitive functioning or slow decline by taking their own proactive actions (04).

Clinicians described *focusing on healthy behaviors* that patients may adopt to increase quality of life and potentially slow the progression of ADRD. This included recommendations such as environmental changes that may make the patient’s life easier, brain games to help with cognition, and the importance of a brain healthy diet. For example, one clinician explained, “I definitely talk about treatment options, but when I talk about treatment options, I tend to focus more on a brain-healthy lifestyle” (12).

Clinicians also discussed *managing patient symptoms* by addressing treatable symptoms or comorbidities: “There are certain symptoms that are very debilitating, that you can get better, depression, anxiety, agitation, poor sleep…Those are all very treatable conditions” (03). Clinicians may do this by recommending pharmacological or non-pharmacological treatments like speech therapy or occupational therapy: “I talk about medical treatments for it. And then I talk about things that need to be done to reduce their disability… . if they need additional rehabilitation, I send them to that individual” (11).

#### Providing patient and family education

Fifth, clinicians described several communication practices used to provide patient and family education about the diagnosis. These included explaining underlying causes of dementia, leaving time during the appointment to answer questions, providing education materials or resources, and following up after the diagnosis appointment.

Clinicians described *explaining underlying causes of dementia* as a way to educate patient and family members due to possible confusion regarding the causes of dementia. For example, patients may be unaware of diseases besides AD that can cause dementia and may conflate other types of dementia with AD: “I often have to educate them that Alzheimer’s disease is a form of dementia, but there are many different forms of dementia” (03). Other clinicians explained that patients sometimes think AD is a worse diagnosis than receiving a general diagnosis of dementia when patients or family members do not understand the etiology of dementia.

Clinicians referenced *leaving time during the appointment* to answer patient or family questions regarding the diagnosis. This allowed for clinicians to better understand patient and family member’s informational and educational needs. For instance, one clinician explained, “I do my best to make clear that when I’m through with the diagnosis, that it’s time for a fairly freewheeling dialogue, they can ask whatever they want, and I try to answer that” (14).

Clinicians talked about *providing educational materials* as another way to provide information about ADRD. This included recommended certain websites to find more information (e.g., Alzheimer’s Association website), as well as providing printed materials to take home, such as pamphlets and information sheets. As one clinician said, “I give them a folder that contains the report, my business card, and then a lot of educational materials, also how to reduce risk factors, how to improve cognitive health and brain health, and education” (13).

Clinicians also described the importance of *following up after the diagnosis* appointment with the patients and/or their family members. Although most clinicians described scheduling routine follow-up appointments three to six months after the diagnosis is given, some clinicians described reaching out to the patient or family members shortly after the diagnosis so patients can process the diagnosis before making decisions or taking proactive steps (e.g., advanced care planning). For example, one clinician described their approach as such:

At that point, at those appointments, I usually don’t go in as hard, because most of the time, most families are just soaking in this information. So, it’s really more of a support, answering any initial questions that they may have. And then usually maybe like a week later, I will have a follow phone call with them. And that’s when we start to develop, “Okay, what now, what do we need to do? Do you have any additional questions?” (07)

### Learning to give a diagnosis

Two major themes emerged to address the second research question regarding how clinicians learned to give a dementia diagnosis: (1) learning through formal training; and (2) learning through self-taught methods. Clinicians who reported using self-taught methods also described learning through formal training methods, but felt they needed more education on how to deliver a diagnosis effectively.

#### Learning through formal training

Clinicians often learned how to communicate a dementia diagnosis through formal training, most commonly by observing mentors or attending clinicians during internship, residency, or fellowships, as well as through continuing medical education. For example, one clinician described their time as a resident as such: “We paid attention to our mentors, watched how they dealt with the patients, and learned our lessons from that and sought to emulate them” (14). Only one clinician referenced learning to break bad news as a significant part of their training, although this was not specific to giving a dementia diagnosis:

Most of the techniques that I use, I learned during fellowship. Part of care, breaking bad news is a significant component of the training. And the diagnosis of dementia, I don’t think there’s anything about it that doesn’t fit really well with the typical breaking bad news sort of structure. I will loosely follow the SPIKES model for breaking bad news. That’s something that I practiced the most and learned the most in fellowship. (12)

#### Learning through self-education

Though less common, some clinicians discussed learning to give a dementia diagnosis through self-taught methods. This included learning through doing: “Learning is lifelong. And in general, you learn by your interactions with patients” (11). Others sought out their own resources to learn about communication practices that can be used to deliver a diagnosis, such as searching for medical education books that covered communicating a diagnosis, as well as searching for online materials like videos. A clinician said: “I did look up on YouTube… I just looked around at different sites just to see what different people did. I’ll be honest, that was actually helpful. It gave me some ideas on ways that I could do it” (10). Reasons for seeking out their own resources to learn to give a diagnosis included not feeling properly trained through their medical education, internships, residency, or fellowships.

## Discussion

For this study we interviewed 15 Florida-based clinicians with a diversity of backgrounds, including gender, race/ethnicity, specialty, and setting (e.g., academic versus not). When describing approaches utilized to communicate a diagnosis of dementia, participants reported using one or more of the five communication practices thematically identified in the results: (1) patient and family-centered communication; (2) tailoring communication to the patient/family; (3) using deliberate choice in diagnostic terms; (4) positively framing information related to the diagnosis; and (5) providing patient and family education. When these approaches were learned, it was most commonly through formal training (particularly observation of senior clinicians) or self-education (e.g., YouTube videos, practice).

Participants described using *patient- and family-centered communication practices* involving family members, checking patient/family understanding, and using empathic communication. Integrating the individual with dementia and family members into true triadic communication can be challenging, with many encounters simply representing a series of dyadic (clinician-patient, clinician-family) exchanges [20]. Clinicians in our study described trying to include and balance the interaction between the individual and their family members (i.e., the dementia triad). Clinicians described doing this by establishing rapport during the dementia diagnosis disclosure process, which is an important communication practice identified by individuals with cognitive impairment and family caregivers of individuals with dementia [42]. Building rapport can result in greater trust, comfort, and honesty with clinicians, as well as alleviate patient anxiety [43]. Some clinicians in the current study described meeting separately with individuals with dementia and their caregivers. This approach has been endorsed by individuals with cognitive impairment and caregivers [42]. Reasons clinicians in our study gave for not doing this included time constraints or feeling that they were not respecting patient autonomy by meeting with caregivers separately. Interestingly though, some clinicians who described meeting with the individual and family separately only reported asking the family members about their communication preferences for disclosing a dementia diagnosis, whereas other clinicians reported asking both the patient and family members’ preferences. In addition to including caregivers as emotional support for the patient [18], caregivers can also function as key members of the care team for the individual with dementia [42]. However, clinicians need to simultaneously manage the different information needs of the individual with dementia and caregivers/family members and avoid colluding with family members [44, 45]. In other words, clinicians must balance patient and caregiver needs while keeping the focus on the individual and allowing for patient autonomy when possible.

Checking understanding was a common practice reported by clinicians, which is a key part of good communication practices in medicine more generally [46] and in the context of communicating a dementia diagnosis [44]. Clinicians described different ways of checking understanding, such as asking patients to repeat back information communicated by the clinician (i.e., the teach back method), or directly asking the patient if they understood. Clinicians also used empathic communication, which involves understanding how another person is feeling and communicating that understanding [47]. In the current study, clinicians described sensitivity toward the challenges and emotions associated with receiving a dementia diagnosis. In some instances, using empathic communication was described by clinicians as a method to build rapport with the individual and family members. This is consistent with the available guidance for dementia diagnosis disclosure, which suggests that clinicians should explore the meaning of the diagnosis during the encounter and the emotional response [44].

Although *tailoring communication to the patient and family* is an aspect of patient- and family-centered communication practices, it was coded as a separate theme because participants described several behaviors specifically tied to the tailoring process. Some clinicians reported asking individuals with dementia and/or family members about their communication preferences prior to giving the diagnosis. Soliciting preferences for dementia diagnosis disclosure in advance is a recommended strategy [44], but was not described by all participants. Clinicians in the current study also reported tailoring communicating based on patient and family characteristics and their reactions in the moment, such as waiting to observe how individuals react or process the diagnosis. When tailoring toward the patient and family background, clinicians’ considerations included the patient’s cognitive abilities, family support, patient and caregiver educational background, and prior knowledge about dementia as well as elements relating to implications of the diagnosis (e.g., anticipated reaction, stigma). This tailoring is in line with guidance suggesting that clinicians should explore expectations and ideas of the person with cognitive impairment [44, 48], account for prior knowledge and perception of a problem [45, 48], tailor information to patient preferences and ideas [44, 48], and be aware of family relationships [45] during disclosure conversations. Prior research suggests that clinicians commonly tailor dementia disclosure based on patient circumstances including awareness of symptoms, dementia severity, and family support [11, 26].

Some clinicians described tailoring their communication to cultural background. This is an important contribution to the literature, as this area is generally understudied. Some studies suggest that individuals identifying as Hispanic or black have missed or delayed dementia diagnoses and are often diagnosed at later stages (e.g., with worse cognitive function and more functional impairments) compared to individuals who identified as non-Hispanic white [49]. Additionally, while fewer than half of individuals with dementia reported being told their diagnosis by a physician regardless of background, proportions were lower for Hispanics and blacks than non-Hispanic whites [50]. Reasons for this are likely complex and include a lack of guidance on how to disclose a dementia diagnosis in a manner that is culturally relevant in different racial and ethnic groups. Future research should expand upon how the patient/family background, culture, and languages of the participants in the triad may impact dementia diagnosis disclosure and how to adapt communication practices can be tailored to be more to be more culturally relevant.

Some clinicians–including both non-specialists and specialists–reported *using deliberate choice in diagnostic terms* during the disclosure process by using general terms such as “dementia” or “memory disorder” or specific terms (e.g., “Alzheimer’s disease”). Reasons for using general terms included the clinician not feeling they had seen the patient long enough to give a more specific diagnosis, either not having enough time in the clinical encounter or not having a long-term relationship with the patient. Further reasons included lack of expertise, not wanting to distress or confuse the patient, or concerns regarding stigma. Irrespective of chosen vocabulary, clinicians noted confusion surrounding dementia and AD terminology, as well as the challenge that definitive diagnosis isn’t currently possible in life. Some clinicians advocated for use of specific language regarding dementia etiology–while acknowledging limitations in current diagnostic approaches–to reduce patient and family uncertainty and connect them to appropriate resources. The mixed approaches in the current study add to the existing literature on why clinicians may use certain language (i.e., general or specific). For instance, a 2019 systematic review suggesting that both specialists and non-specialists use euphemistic terms such as “memory problems” or use the term “dementia” without saying “Alzheimer’s” [11]. Often this was driven by desiring to avoid stigma, loss of hope, or psychological distress [11]. Notably, guidance on dementia disclosure advises that clinicians should explicitly name the diagnosis [44, 48]. This recommendation for explicitly naming the diagnosis can help individuals with dementia and their family plan for the future and access appropriate treatments [7, 44]. A specific, clear diagnosis can also improve quality of care, allowing physicians to “better manage an individual’s comorbid conditions and avoid prescribing medications that may worsen cognition or function” [7]. Family members want to know what an individual with dementia may experience in the future, beyond the diagnosis, and how this may impact them as caregivers [2]. The juxtaposition of reasons to give a specific diagnosis with clinician reluctance also underscores the importance of soliciting patient and caregiver disclosure preferences (also described in our theme *tailoring communication to the patient and family*) so that discussions are patient-driven rather than driven by clinician preferences [44].

Study participants highlighted the importance of *positively framing information about the dementia diagnosis* and described doing so by instilling hope, focusing on healthy behaviors, and managing symptoms to improve or maintain the patients’ quality of life. Our findings regarding positively framing the diagnosis are consistent with approaches reported in other studies (“putting a positive spin or frame on information, such as by focusing the discussion on treatments and services”) [11], as well as suggested disclosure approaches like fostering hope [44], focusing on remaining function [48], and reframing the disease as a challenge of achieving the best life possible despite limitations [48].

The last theme described using communication *to educate patients and families about the dementia diagnosis*, including explaining the underlying dementia cause, allotting appointment time to answer questions, providing educational resources, and following up after the diagnosis appointment. These practices are consistent with available guidance, including educating patients and caregivers regarding the diagnosis and how symptoms relate to an underlying brain disease [45, 48, 51], how the disease will affect the person in the future (prognosis) [44, 45, 48], what pharmacologic and non-pharmacologic treatment options are available [44, 45, 48], realistic expectations for treatment [48], where to find support [44, 45, 48, 51], and directing the caregivers to available resources [48]. However, providing education must be balanced with not giving too much information in one session [44, 48]. This underscores the importance of scheduling follow up visits for additional discussion after disclosure [45, 51] and/or using multiple visits for the dementia disclosure process [44, 48, 51].

Lastly, participating clinicians reported that they learned how to give a dementia diagnosis through formal training or self-taught methods, such as through gaining clinical experience or independently seeking training resources like YouTube videos. Although formal training was most common, this was usually observational in nature (e.g., observing mentors in the disclosure role). Only one clinician described learning formal techniques to help deliver difficult diagnoses, and reported adjusting the SPIKES approach for breaking bad news [52] to disclose a diagnosis of dementia.

### Summary approaches for disclosing a dementia diagnosis

Study participants largely adhered to the approaches consistent with published guidance for disclosing a dementia diagnosis (Table 2) [44, 45, 48]. These were consensus-based and published over ten years ago (2007–2010), but they remain largely applicable to practice, with the notable caveat that they provide no guidance on how to incorporate and utilize culturally sensitive approaches to communicate a dementia diagnosis to diverse populations.

**Table 2:** Approaches for communicating a dementia diagnosis.

Communication Category	Specific Suggested Practices
Preparation	• Identify who the person with cognitive impairment wants to be present
	• Educate regarding the assessment process and possible reactions
	• Plan a disclosure meeting
	• Arrange post-diagnosis support
	• **Establish rapport**
	• **Elicit preferences for disclosure**
Setting	• Arrange quiet, comfortable environment
	• Schedule ample uninterrupted time
Participants	• Limit number of individuals
	• **Involve appropriate family members**
	• Have clinician viewed as the most credible source of information give the diagnosis; clinician should also have established relationship with patient and family
Standard communication practices to employ	• Make eye contact
	• Use verbal and non-verbal communication
	• Use active listening
	• Speak slowly and clearly; use short sentences and simple words
	• Discuss one message/idea at a time
	• Give the person with cognitive impairment time to understand and respond
	• **Check understanding**
Dementia-specific approaches	• **Explore expectations and ideas of the person with cognitive impairment**
	• **Account for prior knowledge and perception of problem**
	• **Tailor information to patient preferences and ideas**
	• **Be aware of family relationships**
	• **Involve and communicate directly with the person with cognitive impairment**
	• **Explicitly name the diagnosis**
	• **Explore the meaning of the diagnosis**
	• **Explore the emotional responses of patient, caregiver**
	• **Foster hope; focus on remaining function and reframing the disease as a challenge of achieving the best life possible despite limitations**
	• Ensure information is consistent across professionals
	• Do not give too much information in one session
Follow-up	• Provide a written summary
	• **Schedule follow up visits for additional discussion**
Topics for discussion	• **Diagnosis and how symptoms relate to underlying brain disease**
	• How the disease will affect the person in the future (prognosis)
	• What pharmacologic and non-pharmacologic treatment options are available; realistic treatment expectations
	• Coping strategies
	• **Where to find support**
	• **Care partner/caregiver health and resources**
	• Future health care wishes (advance care planning)
	• Arrangements for management of financial and personal affairs
	• Safety considerations

Bold: Approaches specifically described by interview participants in the current study.

As mentioned above, one clinician reported adjusting the SPIKES approach for breaking bad news [52] to disclose a diagnosis of dementia. This 6-step format (setting–arrange appropriate location, perception–check with the patient knows, information–ask what the patient wants to know, knowledge–share information in small portions, empathy–expect emotion and respond empathically, strategy/summary–recap important points and outline next steps) covers many of the best practices suggested for dementia disclosure (Table 2). Perhaps unsurprisingly, researchers recently suggested adapting the protocol to create SPIKES-dementia (SPIKES-D) approach [53]. This could be used to facilitate either formal teaching regarding dementia disclosure or self-education, particularly as the SPIKES approach is familiar to many clinicians.

### Strengths and limitations

This study recruited diverse participants in terms of age, gender, and background (e.g., specialty, years in practice), thus reflecting a wide breadth of experiences. Recruitment focused on clinicians working in Florida, which could affect generalizability to other healthcare systems. Most clinicians (n = 11) were affiliated with an academic medical center, which may also impact the generalizability of results. The current study focused on clinician experiences of communicating diagnoses, but ongoing studies will also investigate patient and caregiver experiences of receiving the dementia diagnosis. Furthermore, clinicians self-reported disclosure practices do not imply that these practices are optimal strategies. It is also possible that clinicians provided what they perceived to be socially acceptable answers regarding disclosure communication rather than their typical practices. Future research may benefit from replicating or expanding upon this study in a larger sample, including research questions specifically investigating the impact of cultural differences, and assessing recordings of actual disclosure encounters rather than relying solely on clinician self-report data.

## Conclusion

Florida-based clinicians representing a diversity of backgrounds described dementia disclosure practices largely consistent with prior guidance and approaches to giving bad news more generally [44, 48]. The results of this study highlight the disagreement about optimal approaches to dementia terminology and whether clinicians should use terms such as “Alzheimer disease” during the dementia diagnosis disclosure process. Importantly, not all clinicians mentioned soliciting patient preferences regarding disclosure, so it is likely that some clinicians asked for patient preferences and let them guide the discussion while others based the decision to use (or not use) specific terminology based on clinician preferences or opinions on how the patient and family might respond. The current study focused on clinician self-reported practices and experiences relating to disclosure of dementia diagnoses. A more comprehensive understanding of the diagnosis disclosure process will require elucidating caregiver and family experiences, as well. Additionally, interviews with clinicians caring for under-represented groups suggested that culture/ethnicity can have an impact on the diagnosis disclosure process and on how the patient and their family understand and receive the diagnosis [54–56]. Therefore, more research is needed regarding this finding and best practices for individuals from different backgrounds. Further research in this area is warranted, with the goal of improving dementia disclosure guidance in ways that address cultural differences, as well as balancing a clear diagnosis with patient preferences for diagnostic communication (e.g., general, or specific language).

## Supporting information

S1 File Semi-structured interview guide. (DOCX)

S2 File COREQ checklist. COREQ 32-item checklist outlining the page where each element of qualitative research is reported.
(DOCX)
