# Supplementary material for: Clinician approaches to communicating a dementia diagnosis: An interview study
Source: PLoS One. 2022 Apr 14;17(4):e0267161. doi: 10.1371/journal.pone.0267161 (PMC9009687; doi:10.1371/journal.pone.0267161)
Supplement: S1 File — (DOCX) [file pone.0267161.s001.docx]

Semi-structured interview guide for healthcare providers about communication surrounding the dementia diagnosis

**Welcome:** Thank you for your willingness to participate in an interview about your experience giving diagnoses of dementia. With these interviews, we hope to better understand the experiences of healthcare professionals when giving a diagnosis of dementia and the barriers and facilitators to successful communication about a dementia diagnosis. We are also interviewing patients and caregivers about their experiences. After analyzing the interviews, we hope to be able to develop best practice standards for communicating a dementia diagnosis that account for your experiences and the challenges that you face.

We will be audio recording the discussion so that we can further review the conversations later. Your responses are anonymous and will not be linked to your name. There are no right or wrong answers, just different points of view. You may choose not to answer any question and we can stop at any time. Do you have any questions about the consent form that you received, or about the study?

**Semi-Structured Interview:**

1. Healthcare professional background: What is your degree/role (e.g. MD/physician, PhD, neuropsychologist, physician assistant, nurse practitioner, other)? What is your specialty (e.g. primary care physician, general neurologist, dementia specialist)? How long have you been in practice? Approximately how many individuals do you diagnose with dementia each year? Approximately how many patients do you currently have with dementia?

2. Diagnosis communication expereince:

a. Think of a patient to whom you have given a dementia diagnosis recently. Could you tell me about that patient and how you went about giving the diagnosis? (Probes: How long had you known and been treating the patient? Who was there with the patient? Was the diagnosis given over one visit or several visits?)

b. Is the experience that you just related to us a typical experience? How have the communication with other patients during diagnosis differed from this one?

c. Do you generally give a general diagnosis of dementia or a specific diagnosis (for example, Alzheimer’s disease, dementia with Lewy bodies, vascular dementia)? If it varies, how do you make the decision of what to tell the patient and family?

d. To whom you generally give the diagnosis to (patient/caregiver)? If it varies, how do you make that decision?

e. How many visits are involved in giving the dementia diagnosis? How do you make that decision?

f. What specific information do you try to provide when you give a diagnois, for example, regarding treatments or prognosis?

g. Are there any specific communication strategies or skills you use in these situations? (e.g., empathy, checking understanding)? How did you learn how to give a dementia diagnosis?

3. Diagnosis Communication Barriers, Facilitators and Recommendations:

a. What do you think are the most significant barriers to good communication about a dementia diagnosis? b. What do you think are the most significant facilitators to good communication about a dementia dianosis?

c. If a new doctor about to give a dementia diagnosis for the first time asked you for advice, what would you say?

4. Demographics: In order to be able to describe the participants in this study, it would be helpful if I could get a little bit of demographic information from you.

- [Make sure specialty and years in practice were recorded above; if not, ask here]
- What is your age?
- How do you describe your gender (e.g. male or female)?
- How would you describe your race and ethnicity?
  - Race: White, Black or African American, Asian, American Indian or Alaska Native, Native Hawaiian or Other Pacific Islander
  - Ethnicity: Hispanic/Latino or not Hispanic/Latino

**5. Ending Question:**

We are coming to the end of the interview. I know we covered a lot today, and I really appreciate your time talking with me. Is there anything else you would like to tell me about your experience giving a diagnosis of dementia?

**Closing:**

Thank you so much for your willingness to share your thoughts with us. When we have finished all our interviews, we will be developing a summary of what people said. Would like to receive a copy? (If yes, ask for preferred address, either email or postal.)

People who participated in the interview can receive a $25 gift card. If you would like this, where should we mail this to you?

After analyzing the views expressed in the interviews, we will be convening a group of different stakeholders to help suggest standards for giving a dementia diagnosis. This group will include individuals with recent dementia diagnoses, caregivers, and healthcare professionals of different types (e.g., specialists and non-specialists) so that different views are involved when creating this guidance. If we need someone to represent your group, would you be interested in hearing more about this opportunity in the future? (If yes, ask for best way to contact them when group is convened.)

Thank you again.
