# Supplementary material for: Clinician approaches to communicating a dementia diagnosis: An interview study
Source: PLoS One. 2022 Apr 14;17(4):e0267161. doi: 10.1371/journal.pone.0267161 (PMC9009687; doi:10.1371/journal.pone.0267161)
Supplement: S2 File — COREQ 32-item checklist outlining the page where each element of qualitative research is reported. (DOCX) [file pone.0267161.s002.docx]

**COREQ (COnsolidated criteria for REporting Qualitative research) Checklist**

A checklist of items that should be included in reports of qualitative research. You must report the page number in your manuscript

where you consider each of the items listed in this checklist. If you have not included this information, either revise your manuscript accordingly before submitting or note N/A.

**Topic**

**Item No.**

**Guide Questions/Description**

**Reported on Page No.**

**Domain 1: Research team and reﬂexivity**

*Personal characteristics*

Interviewer/facilitator

1

Which author/s conducted the interview or focus group?

10

Credentials

2

What were the researcher’s credentials? E.g. PhD, MD

10

Occupation

3

What was their occupation at the time of the study?

10

Gender

4

Was the researcher male or female?

10

Experience and training

5

What experience or training did the researcher have?

10

*Relationship with participants*

Relationship established

6

Was a relationship established prior to study commencement?

9

Participant knowledge of the interviewer

7

What did the participants know about the researcher? e.g. personal goals, reasons for doing the research

9-10

Interviewer characteristics

8

What characteristics were reported about the inter viewer/facilitator?

e.g. Bias, assumptions, reasons and interests in the research topic

9-10

**Domain 2: Study design**

*Theoretical framework*

Methodological orientation and Theory

9

What methodological orientation was stated to underpin the study? e.g. grounded theory, discourse analysis, ethnography, phenomenology, content analysis

8

*Participant selection*

Sampling

10

How were participants selected? e.g. purposive, convenience, consecutive, snowball

9

Method of approach

11

How were participants approached? e.g. face-to-face, telephone, mail, email

9

Sample size

12

How many participants were in the study?

12

Non-participation

13

How many people refused to participate or dropped out? Reasons?

12

*Setting*

Setting of data collection

14

Where was the data collected? e.g. home, clinic, workplace

10

Presence of non- participants

15

Was anyone else present besides the participants and researchers?

12

Description of sample

16

What are the important characteristics of the sample? e.g. demographic data, date

12

*Data collection*

Interview guide

17

Were questions, prompts, guides provided by the authors? Was it pilot tested?

10

Repeat interviews

18

Were repeat inter views carried out? If yes, how many?

9

Audio/visual recording

19

Did the research use audio or visual recording to collect the data?

10

Field notes

20

Were ﬁeld notes made during and/or after the inter view or focus group?

10

Duration

21

What was the duration of the inter views or focus group?

12

Data saturation

22

Was data saturation discussed?

11

Transcripts returned

23

Were transcripts returned to participants for comment and/or

10

11

Developed from: Tong A, Sainsbury P, Craig J. Consolidated criteria for reporting qualitative research (COREQ): a 32-item checklist

for interviews and focus groups. *International Journal for Quality in Health Care*. 2007. Volume 19, Number 6: pp. 349 – 357

**Once you have completed this checklist, please save a copy and upload it as part of your submission. DO NOT include this**

**checklist as part of the main manuscript document. It must be uploaded as a separate file.**

**Topic**

**Item No.**

**Guide Questions/Description**

**Reported on Page No.**

correction?

**Domain 3: analysis and ﬁndings**

*Data analysis*

Number of data coders

24

How many data coders coded the data?

11

Description of the coding tree

25

Did authors provide a description of the coding tree?

11

Derivation of themes

26

Were themes identiﬁed in advance or derived from the data?

11

Software

27

What software, if applicable, was used to manage the data?

Participant checking

28

Did participants provide feedback on the ﬁndings?

10

*Reporting*

Quotations presented

29

Were participant quotations presented to illustrate the themes/ﬁndings? Was each quotation identiﬁed? e.g. participant number

12-22

Data and ﬁndings consistent

30

Was there consistency between the data presented and the ﬁndings?

12-22

Clarity of major themes

31

Were major themes clearly presented in the ﬁndings?

12-22

Clarity of minor themes

32

Is there a description of diverse cases or discussion of minor themes?

12-22
